# GATSol, an enhanced predictor of protein solubility through the synergy of 3D structure graph and large language modeling

**DOI:** 10.1186/s12859-024-05820-8

**Published:** 2024-06-01

**Authors:** Bin Li, Dengming Ming

**Affiliations:** College of Biotechnology and Pharmaceutical Engineering, Nanjing Tech University, 30 South Puzhu Road, Jiangbei New District, Nanjing, 211816 Jiangsu People’s Republic of China

**Keywords:** Protein solubility, Alphafold, ESM-1b, Graph neural network, Attention

## Abstract

**Background:**

Protein solubility is a critically important physicochemical property closely related to protein expression. For example, it is one of the main factors to be considered in the design and production of antibody drugs and a prerequisite for realizing various protein functions. Although several solubility prediction models have emerged in recent years, many of these models are limited to capturing information embedded in one-dimensional amino acid sequences, resulting in unsatisfactory predictive performance.

**Results:**

In this study, we introduce a novel Graph Attention network-based protein Solubility model, GATSol, which represents the 3D structure of proteins as a protein graph. In addition to the node features of amino acids extracted by the state-of-the-art protein large language model, GATSol utilizes amino acid distance maps generated using the latest AlphaFold technology. Rigorous testing on independent eSOL and the *Saccharomyces cerevisiae* test datasets has shown that GATSol outperforms most recently introduced models, especially with respect to the coefficient of determination R^2^, which reaches 0.517 and 0.424, respectively. It outperforms the current state-of-the-art GraphSol by 18.4% on the *S*. *cerevisiae*_test set.

**Conclusions:**

GATSol captures 3D dimensional features of proteins by building protein graphs, which significantly improves the accuracy of protein solubility prediction. Recent advances in protein structure modeling allow our method to incorporate spatial structure features extracted from predicted structures into the model by relying only on the input of protein sequences, which simplifies the entire graph neural network prediction process, making it more user-friendly and efficient. As a result, GATSol may help prioritize highly soluble proteins, ultimately reducing the cost and effort of experimental work. The source code and data of the GATSol model are freely available at https://github.com/binbinbinv/GATSol.

**Supplementary Information:**

The online version contains supplementary material available at 10.1186/s12859-024-05820-8.

## Introduction

Solubility is a significant indicator of protein performance in biotechnology and medicine [1–3], especially for recombinant proteins in disease therapy [4]. For example, recombinant proteins used for disease treatment are usually injected subcutaneously, and they need to have a high solubility to avoid aggregation when they enter the body at high therapeutic concentrations [5]. This is also true in the pharmaceutical field, where one needs to consider increasing the solubility of antibodies to avoid aggregation that could lead to a decrease in their activity in vivo [6]. Standard protein production methods in *Escherichia coli* (*E. coli*) often result in low solubility, hindering their manufacturability [7]. Improving protein solubility experimentally involves using weak promoters [8], lower temperatures [9], modified growth media, or optimizing other expression conditions [10]. However, even today, screening for high solubility through experiments is still time- and effort-consuming, which has led to the development of bioinformatics methods to predict protein solubility over time, thus avoiding many unnecessary wet experimental studies [11].

Over the past decades, several computational methods have been developed to predict protein solubility using various features of protein sequences. Most of these tools with relatively high accuracy use traditional machine learning models such as support vector machines [12] and gradient boosters [13], whose input consists of pre-extracted features, i.e., features extracted from protein sequences by other bioinformatics tools before being fed into the machine learning model. For example, SOLpro employs a two-stage SVM model to train 23 features extracted from protein sequences [10]. PROSO II uses a two-layer structure with a Parzen window [14], whose first layer is a first-stage logistic regression model, and the second layer is a second-stage logistic regression model [15]. More recent models, such as SoluProt, use a new independent test set and utilize the frequencies of significant dimers extracted from protein sequences as part of the input features [16]. DeepSol, an earlier deep learning predictor proposed by Khurana and colleagues [17], was constructed as a single-stage predictor that uses the high-dimensional solubility classification features encoded in the typical amino acid k-mers and their non-linear local interactions extracted from protein sequences, using a parallel convolutional neural network with different filter sizes [18].

However, one-dimensional (1D) information, whether in the form of core amino acids, specific motifs, overall amino acid sequence identity, or hidden Markov models [19], cannot fully illuminate the functional characteristics of proteins. Deep learning models using graphical neural networks, which exploit the contact information of amino acid pairs, such as GraphSol, have also emerged [20, 21]. The advantage of this method is that it adds some physical significance and interpretability to the predictor by considering amino acid residue pair contact information. However, The data processing procedure in the GraphSol model is quite intricate, making it challenging to input custom data for predictions. Additionally, the accuracy of these prediction methods still needs to meet the demands of practical applications. Consequently, there remains ample room for enhancement and improvement in this regard.

Numerous artificial intelligence methods have emerged dedicated to precisely predicting high-resolution 3D protein structures from one-dimensional amino acid sequences. These advancements have enabled us to access the 3D structures of many proteins, leveraging the predictions made by these AI models. Notably, Alphafold has made remarkable strides in protein 3D structure prediction [22–24], earning validation and acclaim through the Critical Assessment of Protein Structure Prediction. This accomplishment has rendered the predicted protein structures efficient for applications in scientific research. Furthermore, research in large-scale language modeling for proteins is booming, with the ESM-1b model emerging as a prominent representative [25, 26]. Based on the Transformer architecture, this is an unsupervised protein language model. The self-attention mechanism of the ESM-1b allows it to compute pairwise interactions between residues in protein sequences directly, capturing the intricate interdependencies and interactions among amino acid residues at different positions. These interactions are intricately linked to the protein's structural characteristics and are reflected in the sequence patterns. What's noteworthy is that unsupervised language models can leverage extensive sequence data from protein databases without the need for manual annotations. The ESM-1b model, trained on a vast dataset comprising 250 million protein sequences, can extract meaningful features directly from amino acid sequences. These feature representations encode crucial information about a protein's secondary and tertiary structures, functionality, homology, and more, which can be intuitively conveyed through linear projections.

This study introduces GATSol, an innovative structure-based solubility prediction method that uses graph-attentive deep learning modeling [27]. In GATSol, proteins are represented by a graph data structure consisting of nodes and edges, where nodes represent amino acids and edges connect nodes whose amino acids are closely contacted in the 3D structure. The method then applies graph neural networks to extract features and learn the underlying relationships in the graph data, a technique that has proven to be very successful in predicting protein properties [28, 29]. Specifically, Alphafold is used to predict the 3D structure of proteins based on their sequences, from which amino acid distance maps are generated. The protein large language model, ESM-1b, is applied to the sequence to extract embedding features for each node in the protein graph. The model was then fully trained and rigorously tested using the eSOL database. For a fair comparison, we also applied the model to a larger test set of *S*. cerevisiae to assess its robustness and applicability. Our calculations revealed GATSol outperformed recently introduced models in the two test datasets.

## Methods

### Dataset

During model training and testing, we utilized two datasets: eSOL and *S. cerevisiae*. The eSOL dataset underwent preprocessing to remove intra-dataset homology. Subsequently, it was split into a training set and the first independent test set. To ensure data diversity, the *S. cerevisiae* dataset was compared to eSOL to identify and remove homologous sequences, forming the second independent test set. The distribution of sequence lengths and solubilities of the proteins used for training and testing is shown in Fig. S1 [see Supplementary file 1]. It can be observed that the solubility distribution in the dataset exhibits a trend where shorter protein sequences tend to have relatively higher solubility from Fig. S1A, S1B, and S1C, with this trend being more pronounced in the eSol dataset for training. The specific dataset treatments are described below.

### eSOL dataset

eSOL is a database on the solubility of the entire ensemble *of E. coli* proteins individually synthesized by the PURE system*,* which is a chaperone field [30]. To create the training set, we utilized the eSol database and adhered to the same data processing of GraphSol. Initially, the eSol dataset comprised 4132 proteins, with solubility defined as the ratio of supernatant grade to total grade in the PURE physical chemistry experiment [31]. These 4132 proteins were initially mapped to the NCBI database using their gene names, resulting in 3144 samples.

Minimizing homology within and between the training and test sequences was imperative to ensure a fair evaluation of the prediction tool's performance. Conventional clustering tools such as CD-HIT, MMseqs 2, and BLASTCLUST were found to need to be more sufficient in achieving this homology separation [32]. Therefore, we introduced our ggsearch36 tool [33], which allows for global identity comparisons, and employed the following homology separation procedure:

1. Prepare a dataset in FASTA format for homologation.
2. Extract the first amino acid sequence from the dataset to initialize a library file.
3. Retrieve the second protein sequence from the dataset and compare homology with the library file using ggsearch36. The sequence is added to the library file if the global identity between the two sequences is < 30% and the E-value is ≤ 1e−6.
4. Repeat the above step for the third protein sequence and continue until all data in the dataset have been homologously compared once.

The final library file represents the homology-separated dataset, which included a total of 2679 protein sequences. To ensure equitable comparisons with other models, we adopted the same training set division strategy as GraphSol. Any amino acid sequences presented in GraphSol's training set were included in our training set, while the remainder were allocated to the test set. As a result, the final dataset consists of 2019 amino acid sequences assigned to the training set (75%) and 660 amino acid sequences assigned to the test set (25%). It is worth noting that both the training and test sets utilized in our model constitute equal-sized subsets of the corresponding training and test datasets of GraphSol. This equivalence facilitates a direct and insightful comparative analysis between our model and GraphSol.

### *Saccharomyces cerevisiae* dataset

For independent external testing, we employed a unique protein dataset sourced from *S. cerevisiae* [34]. Initially, the dataset comprised 447 proteins with UniProt Entry identifiers and their corresponding solubility information obtained through the PURE cell-free expression assay. To ensure the integrity of the dataset, we strictly filtered internal homologies using the same meticulous process as the eSOL dataset. After removing internal homology, the dataset was streamlined to 414 entries.

We then performed an exhaustive homology removal process between this dataset of 414 entries and the eSOL dataset. Through these rigorous procedures, we obtained the *S. cerevisiae* dataset—*S. cerevisiae*_368, which currently contains 368 amino acid sequences. In addition, we isolated a subset of the *S. cerevisiae* dataset—*S. cerevisiae*_108 [20], which includes 108 proteins, mirroring the *S. cerevisiae* dataset used by GraphSol. This was done to facilitate direct comparisons with other models.

## Protein graph

### Node features

Nodes play a pivotal role in the graph structure [35]. In the protein graph, each amino acid is considered a separate node, and we extract features from each amino acid in the sequence as node attributes. We tested the ESM-1b and Blosum62 features, did ablation experiments on both features separately, and found that the model works best when these features work together. This suggests that although the ESM-1b feature contains a large amount of information about the structure of biomolecules, the Blosum62 feature, as a widely used one, can still complement the enhancement effect.

### ESM-1b features

The ESM-1b model is a cutting-edge transformer network honed by self-supervised learning of an astonishing dataset of about 250 million proteins from the UniRef50 dataset [25]. This model takes amino acid sequences as input and generates numerical representations of these sequences, often called protein embeddings. A key strategy throughout the training of ESM-1b was to randomly mask approximately 15% of the amino acids in the protein sequence. Subsequently, the model was fine-tuned to identify and predict the identities of these masked amino acids. This rigorous training regimen forces the model to encapsulate local and global protein sequence information into a 1280-dimensional representation vector assigned to each amino acid. Thus, the ESM-1b model can represent each amino acid in an amino acid sequence of length L as a 1280-dimensional vector, yielding an $\text{L}\times 1280$-dimensional feature matrix.

### Blosum62 features

Blosum62 stands for BLOcks of Amino Acid SUbstitution Matrix 62, a substitution matrix used to evaluate the possibility of amino acid substitutions during protein sequence comparisons [36]. This matrix utilizes a large amount of comparative data from known protein sequences to derive substitution scores between different amino acids. This is achieved by analyzing the substitutions of alternative amino acids at different positions. Thus, Blosum62 encodes each amino acid in a protein sequence into a 20-dimensional feature vector that reflects their respective substitution propensities. The functionality for generating the corresponding $\text{L}\times 20$ Blosum62 matrix from the raw amino acid sequence of L length has been seamlessly integrated into iFeatureOmega, and we will leverage this program to generate the Blosum62 matrix [37] directly. Notably, Blosum62 frequently performs excellently in protein-related tasks [37].

### Distance map

In the intricate world of protein structures, the spatial relationships between amino acids, especially the proximity between α-carbon atoms, play a pivotal role. Protein backbone structures based on α-carbon atoms have achieved leading results in protein generation [38]. These distances reveal the three-dimensional configuration of proteins. We construct the adjacency matrix, also known as the distance map, by precisely measuring and recording these distances in the protein graph.

Obtaining the 3D structure of a protein directly from its amino acid sequence is typically challenging. Here, for a given amino acid sequence, we generated its 3D structure by modeling with Alphafold [22] and saved it as a PDB file. This allows us to calculate the distances between the α-carbon atoms of individual amino acids and thus determine the distance map between residues.

In our protein graph representation, each amino acid corresponds to a node, and we use these distances to determine whether edges exist between different amino acid pairs to construct a distance map. When the distance between the α-carbon atoms of two amino acids falls below a predefined threshold, we build an edge to indicate interaction or proximity. In this way, by continuously adding edges to the amino acid nodes, we ended up with a distance map that characterizes the structure of the protein. To determine the most suitable threshold, we used a cross-validation method.

**Fig. 1:**
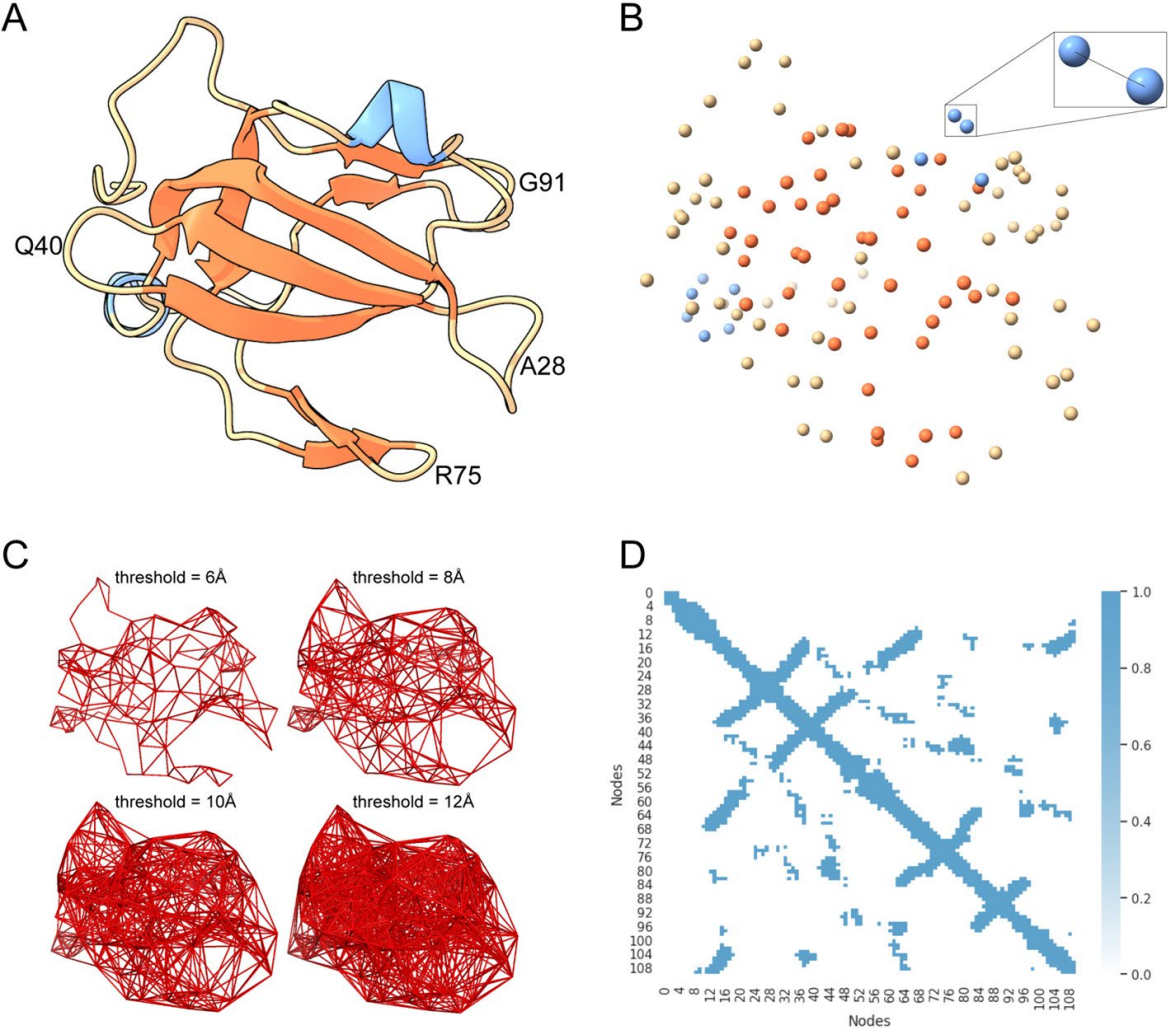
**A** 3D structure of the amino acid sequence corresponding to ygjH predicted with Alphafold in the eSOL data, the blue part represents the α-helix structure, and the orange part represents the strand structure; **B** Atom map of the ygjH protein in Fig. A after retaining only the Cα, and the upper right corner is the example of the calculated distances by taking two Cαs; **C** Adding edges to all Cα atom pairs according to the distance thresholds of 6Å, 8Å, 10Å, and 12Å, respectively; **D** Showing the distance map of α-carbons under the distance threshold of 10Å

An example of a distance map is given in Fig. 1, in which ygjH is a nucleic acid-binding protein in the eSOL dataset. The figure shows several bands that cross the main diagonal, which occur at β-turn positions, including amino acids A28, Q40, R75, G91, etc. Branches approximately parallel to the main diagonal correspond to many residues parallel to each other in the 3D structure, forming either a β-sheet structure or an α-helix/β-sheet folded structure. Extracted distance maps record surface information that may reflect many features of protein structure and solubility properties.

### Neural network architecture

**Fig. 2:**
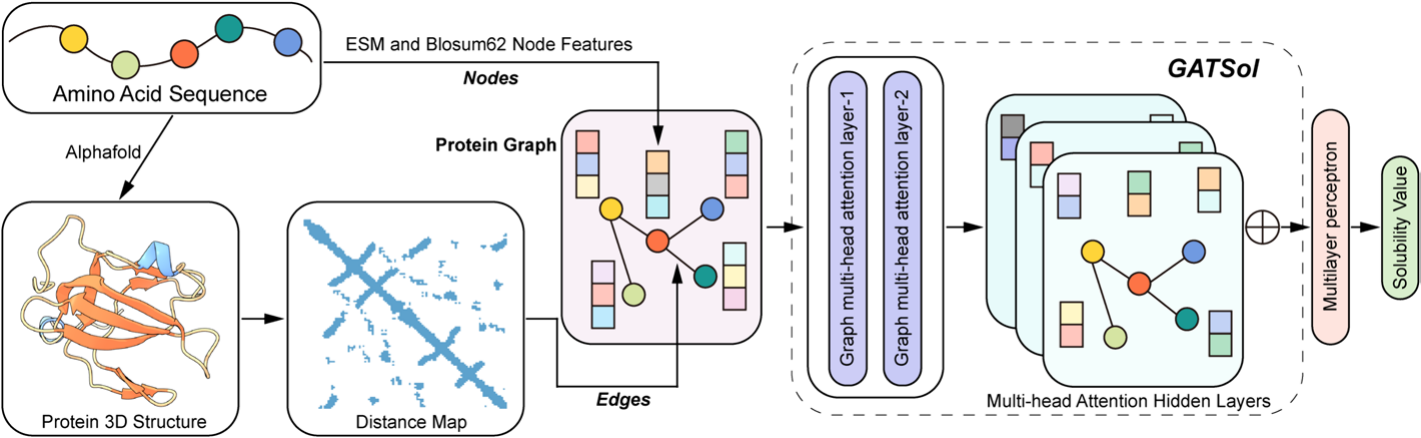
The overall framework of the GATSol model consists of several key components. First, the node features of the protein graph are carefully extracted from amino acid sequences. Second, the graph's edges are derived from the protein structure modeled by Alphafold and based on amino acid sequences. After two iterations of the graph multi-head attention layers, the features from the multi-head attention hidden layers are meticulously integrated. These integrated features are the input to the multilayer perceptron for predicting solubility values

The Graph Attention Network (GAT) employs an attention mechanism that assigns weights to neighboring node features and re-aggregates these features [27]. Importantly, these weights are determined entirely by the characteristics of the node features, independent of the underlying graph structure. The GATSol model autonomously extracts feature information from nodes and edges as the protein graph iteratively propagates node information. This process assigns different weights to nodes in the intermediate hidden layers to create fixed-size graph representation vectors. These vectors are then subsequently consolidated via fully connected layers. The overall architecture of the model is shown in Fig. 2.

## Graph attention networks

Following the notation of reference [27], for a protein consisting of *L* amino acids, the input of node feature can be written as $\mathbf{h}={\overrightarrow{\{h}}_{1},{\overrightarrow{h}}_{2},\dots ,{\overrightarrow{h}}_{L}\},{\overrightarrow{h}}_{i}\in {\mathbb{R}}^{F}$, where ${\overrightarrow{h}}_{i}$ is an amino-acid dependent *F*-dimensional f feature vector, and in this work $F=1300$, obtained by summing ESM-1b features in 1280 dimensions and Blosum62 features in 20 dimensions. Denote $\mathbf{A}\in {\mathbb{R}}^{L\times L}$ as the adjacency matrix representing the neighboring contact information. The primary function of the attention layer is to generate an output set of node features ${\mathbf{h}}^{\mathbf{^{\prime}}}=\left\{{\overrightarrow{h}}_{1}{^\prime},{\overrightarrow{h}}_{2}{^\prime},\dots ,{\overrightarrow{h}}_{N}{^\prime}\right\}$, $\overrightarrow {h}_{i}{^\prime} \in {\mathbb{R}}^{{F^{\prime}}}$, based on input $\mathbf{h}$ and $\mathbf{A}$. The dimensionality of the feature vectors may be changed during the process: $F \to F^{\prime }$.

To retain enough expressive power, at least one learnable linear transformation is required. For example, transfer from the dimension A features ${\overrightarrow{h}}_{i},{\overrightarrow{h}}_{j}$ to the new dimension B features ${\overrightarrow{h}}_{i}{^\prime},{\overrightarrow{h}}_{j}{^\prime}$ for nodes $i,j$, and then we leverage self-attention to allocate attention weights to each node, ${e}_{ij}=a(\mathbf{W}{\overrightarrow{h}}_{i},\mathbf{W}{\overrightarrow{h}}_{j})$, where $a$ signifies a shared attention mechanism: ${\mathbb{R}}^{{F^{\prime}}} \times {\mathbb{R}}^{{F^{\prime}}} \to {\mathbb{R}}$, which used to calculate the attention factor ${e}_{ij}$, the importance of node *j*’s features to node *I*’s, and $\mathbf{W}\in {\mathbb{R}}^{{F}^{;}\times F}\text{ is a weight matrix which parametrizes a shared linear transformation}$.

When evaluating the target node $i$, we used masked attention to specifically compute the correlation ${e}_{ij}$ among nodes $j\in {\mathcal{N}}_{i}$ within its neighborhood, encompassing the target node's own influence, where ${\mathcal{N}}_{i}$ is some neighborhood of node i in the graph. To ensure a balanced distribution of weights among various nodes, we employ softmax normalization to normalize the computed correlations with all neighboring nodes uniformly:

$$\alpha_{ij} = {\text{softmax}}_{j} \left( {e_{ij} } \right) = \frac{{\exp \left( {e_{ij} } \right)}}{{\sum\nolimits_{{k \in {\mathcal{N}}_{i} }} {\exp } \left( {e_{ik} } \right)}}$$

when it comes to selecting the $a$ parameter, there are two viable approaches. One option is to use the inner product of vectors to establish an unparameterized form of correlation computation. Alternatively, $a$ can be defined as a parameterized neural network layer, provided it fulfills the requirement of producing a scalar value signifying the correlation between the two elements. In this context, $a$ represents a single-layer feed-forward neural network, parameterized by the weight vector $\overrightarrow {{\text{a}}} \in {\mathbb{R}}^{{2F^{\prime}}}$, and uses LeakyReLU as the activation function with a negative semiaxis slope of 0.2. Let the symbol $\parallel$ denote the concatenation operation that merge two weighted feature vectors $\mathbf{W}{\overrightarrow{h}}_{i}$ and $\mathbf{W}{\overrightarrow{h}}_{j}$ into a new feature vector, and then compute the complete weight coefficients using the following equation:

$${\alpha }_{ij}=\frac{\text{exp}(\text{LeakyReLU }({\overrightarrow{\mathbf{a}}}^{T}[\mathbf{W}{\overrightarrow{h}}_{i}\parallel \mathbf{W}{\overrightarrow{h}}_{j}]))}{\sum_{k\in {\mathcal{N}}_{i}}\text{exp}(\text{LeakyReLU }({\overrightarrow{\mathbf{a}}}^{T}[\mathbf{W}{\overrightarrow{h}}_{i}\parallel \mathbf{W}{\overrightarrow{h}}_{k}]))}$$

After obtaining the normalized attention coefficients, we proceed to compute a linear combination of their associated features. Subsequently, by means of a nonlinear activation function $\sigma$, we obtain the final output feature vector for each node:

$$\vec{h}_{i}^{^\prime } { = }\sigma \left( {\sum\limits_{{j \in {\mathcal{N}}_{i} }} {\alpha_{ij} } {\mathbf{W}}{\vec{\text{h}}}_{{\text{j}}} } \right)$$

### Multi-head attention

We employ multi-head attention to enhance the stability of the self-attention learning process. In other words, we invoke K sets of mutually independent attention mechanisms according to the initial formula, and then combine the resulting outputs:

$${\overrightarrow{h}}_{i}{^\prime}={\parallel }_{k=1}^{K}\sigma (\sum_{j\in {\mathcal{N}}_{i}}{\alpha }_{ij}^{k}{\mathbf{W}}^{k}{\overrightarrow{h}}_{j})$$

where $\parallel$ is the concatenation operation and ${\alpha }_{ij}^{k}$ is the weight coefficients computed by the k set of attention mechanisms ${\mathbf{W}}^{k}$ is the corresponding input linear transformation matrix, and the final output node eigenvector ${\overrightarrow{h}}_{i}{^\prime}$ contains the ${\text{KF}}{^\prime}$ dimension features.

### Multilayer perceptron

The vectors resulting from the fusion of multiple attention mechanisms are passed into a multilayer perceptron [39]. This perceptron serves the purpose of generating the predicted solubility $S$ that we seek:

$$S=\text{ReLU}({{\varvec{w}}}_{{\varvec{M}}{\varvec{L}}{\varvec{P}}}{{\overrightarrow{h}}_{i}{^\prime}}^{\text{T}}+\text{b})$$

where ${{\varvec{w}}}_{{\varvec{M}}{\varvec{L}}{\varvec{P}}}\in {\mathbb{R}}^{{\text{KF}}{^\prime}}$ is the hidden layer weights of the multilayer perceptron and b is the hidden layer bias.

### Training and evaluation

#### Five-fold cross-validation

K-fold cross-validation is a widely employed technique for evaluating machine learning models [40]. It evaluates model performance, reduces overfitting, and facilitates parameter selection and tuning. This approach enhances the model's generalization ability and ensures superior performance on previously unseen data.

In this study, we performed five-fold cross-validation on the training dataset, which was not used in the independent tests. Specifically, the protein dataset designated for training was randomly divided into five subsets. Each iteration of training rotates four of these subsets for model training, and the remaining one is used for model evaluation. This process was iterated five times, and the hyperparameter optimization was performed using the average coefficient of determination (R^2^) value reflecting the model's performance over the five rounds. After fine-tuning the optimal hyperparameters, the model was trained using the entire training dataset and tested independently on a separate test dataset.

### Evaluation indicators

When training the GATSol model, we used the root-mean-square error as the loss function. We utilized R^2^ to evaluate the model's performance while fine-tuning the hyperparameters. In addition, for comparison with the solubility classifier models reported in the recent literature, we categorized all predicted protein solubility values into two groups: i.e., those greater than or equal to the 0.5 thresholds are labeled as soluble and the rest as insoluble. We evaluated the model's performance using the area under the Receiver Operating Characteristic (ROC) curve (AUC), accuracy, precision, and recall, as outlined in the following formula [41]:

$$\text{Accuracy}=\frac{\text{TP}+\text{TN}}{\text{TP}+\text{TN}+\text{FP}+\text{FN}}$$

$$\text{Precision = }\frac{\text{TP}}{\text{TP}+\text{FP}}$$

$$\text{Recall = }\frac{\text{TP}}{\text{TP}+\text{FN}}$$

$${\text{F}}_{1}=\frac{2\times ({\text{Precision}}\times \text{Recall})}{({\text{Precision}}+\text{Recall})}$$

where TP represents true positives (soluble proteins), FP stands for false positives (non-soluble proteins predicted as soluble), TN represents true negatives, and FN stands for false negatives.

## Results and discussion

### Node feature and distance map threshold selection

**Fig. 3:**
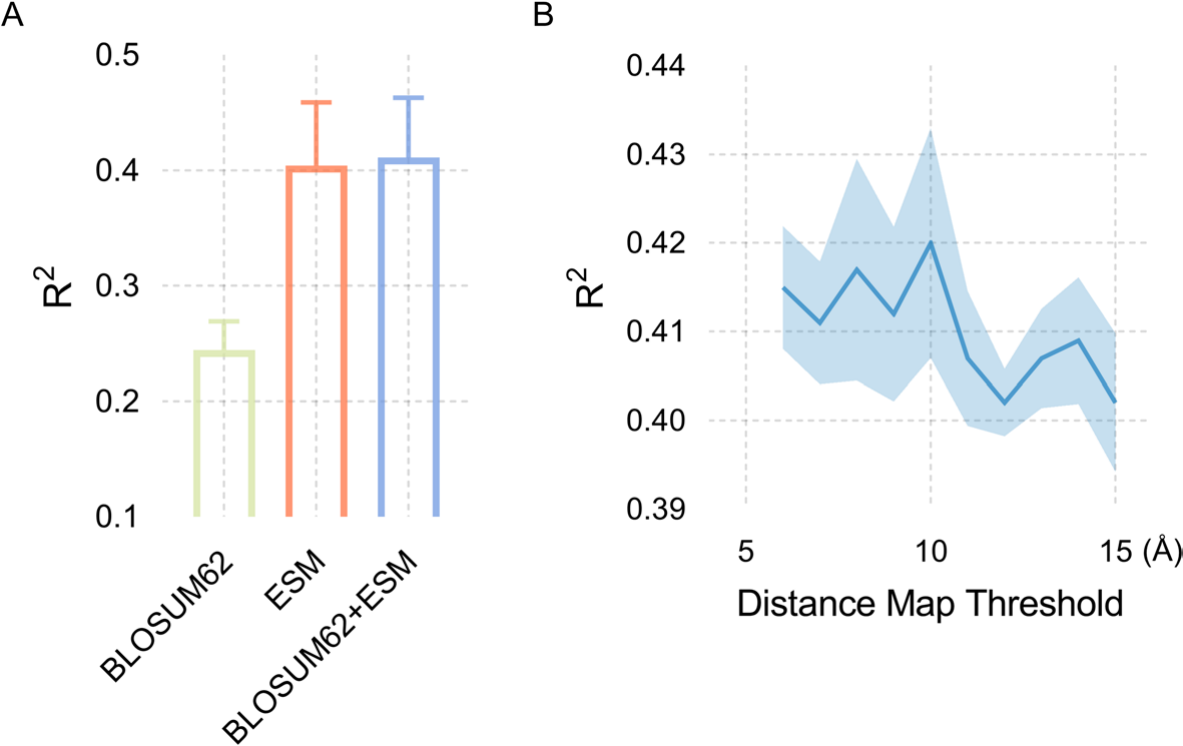
**A** Results of ablation experiments with amino acid node feature selection; **B** Results for distance thresholding of edges between nodes in a protein graph

For our ablation experiments, we conducted five-fold cross-validation on the training set to meticulously select the optimal node feature combinations and determine the most effective threshold for the distance graph. As depicted in Fig. 3A, our model attains its peak performance, with an impressive R^2^ value of 0.411, when ESM-1b and Blosum62 features are seamlessly integrated into the model. This achievement underscores our model's aptitude for effectively harnessing the combined information from both feature sets. Notably, the 20-dimensional Blosum62 features are a valuable complement to the already information-rich 1280-dimensional ESM-1b features.

As shown in Fig. 3B, in our quest to ascertain the optimal threshold for the distance map, it became evident that 10Å stood out as the superior choice. This selection enables our model to achieve the highest R^2^ value, emphasizing its proficiency in fine-tuning model parameters for peak performance.

### Hyper-parameter optimization

**Fig. 4:**
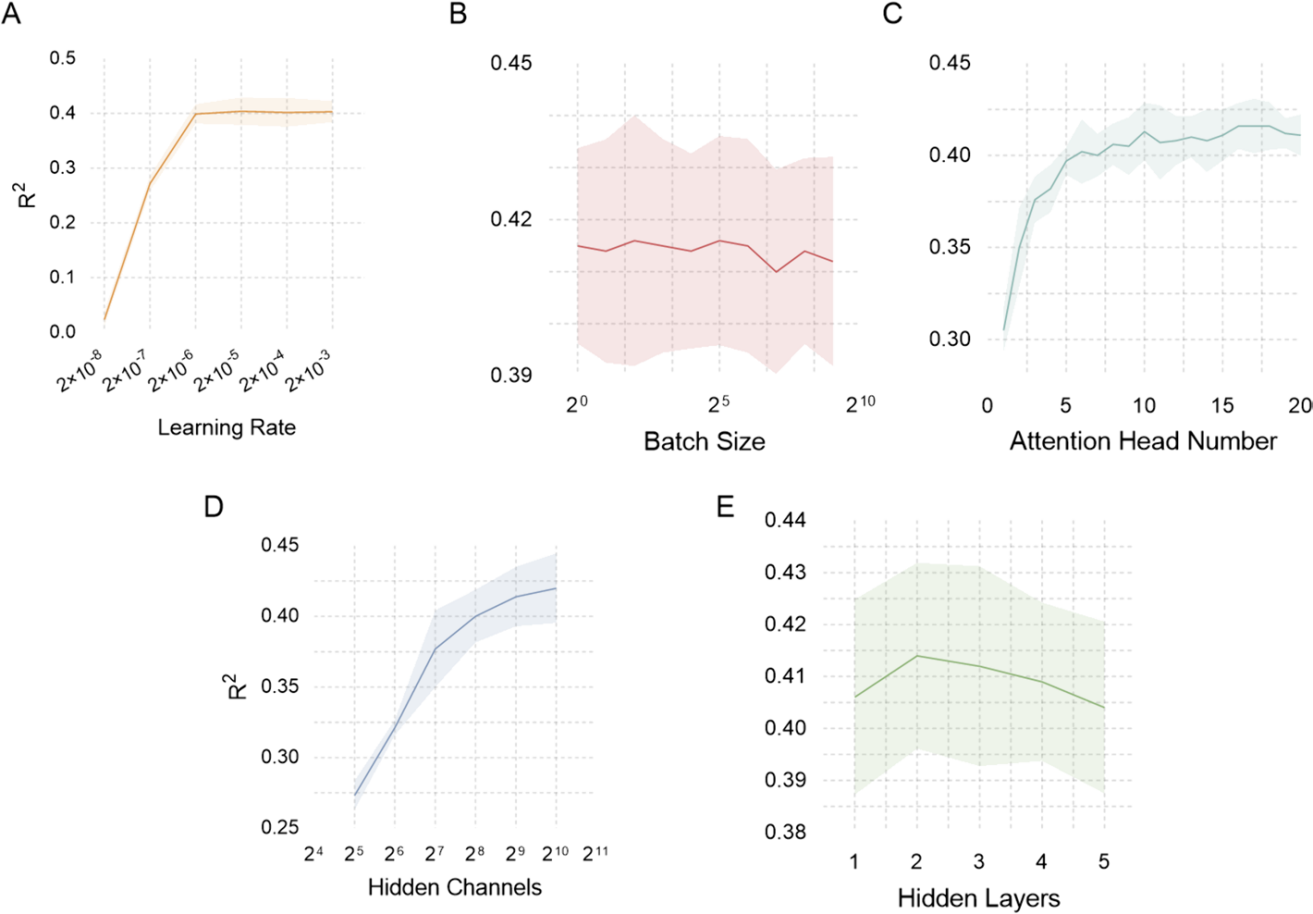
Hyper-parameter optimization process of learning rate (**A**), batch size (**B**), number of attention heads (**C**), hidden channel count (**D**), and hidden layers (**E**)

We conducted five-fold cross-validation on the identical training set to fine-tune the model's hyperparameters separately, including the learning rate, batch size, number of attention heads, hidden channel count, and hidden layers, as shown in Fig. 4 below.

During the five-fold cross-validation, we observed a noteworthy pattern in the model's fitting performance, typically characterized by reaching a peak or encountering a bottleneck as hyperparameters were adjusted. As a general guideline, selecting a minimal learning rate can result in sluggish convergence or getting stuck in local optima, while opting for an overly large learning rate may induce instability or even divergence in the training process. Similarly, a batch size that is too small can lead to a disproportionate increase in model training time or heightened gradient estimation noise, thereby amplifying training instability. Conversely, a considerable batch size can exponentially escalate the model's memory demands during training and elevate the risk of overfitting, compromising the model's ability to generalize effectively.

We meticulously determined the model's hyperparameters to balance achieving optimal results and avoiding undue escalation in model complexity and computational costs. Specifically, we arrived at the following settings: a learning rate of 2 × 10^–6^, a batch size of 4, 16 attention heads, 1024 hidden channels, and two hidden layers. These parameter choices were instrumental in fine-tuning the model for its best possible performance.

### Comparisons with other predictors

We conducted a comparative analysis of our model against state-of-the-art methods. Recognizing that model ensemble significantly amplifies model complexity and computational overhead, we opted not to engage in model ensemble, thereby ensuring a basis for practical usability.

As depicted in Table 1, when benchmarking the GATSol model against other existing methods, it's worth noting that most of these methods were initially designed for predicting discrete states. We transformed the problem into a binary classification task to facilitate a fair comparison, aligning with GraphSol's approach. We employed a threshold value of 0.5 to categorize proteins as soluble, and our results underscored significant superiority in key performance metrics. Moreover, we used the coefficient of determination R^2^ to comprehensively evaluate the model's regression ability, providing a broader assessment of its performance. The GATSol model demonstrated an R^2^ value of 0.517, an Accuracy of 0.791, a Precision of 0.781, a recall of 0.745, an F1 Score of 0.763, and an AUC of 0.882. These values surpass those of the GraphSol model using a Graph Neural Network, and the R^2^ value exceeds GraphSol's by 7.0%, also having a 3.2% advantage over GraphSol (Ensemble). This disparity underscores our model's proficiency in discerning latent data characteristics essential for accurate proteolysis prediction.

**Table 1:** Performance comparison of GATSol with existing protein solubility predictors on the independent eSOL test dataset

Models	R^2^	Accuracy	Precision	Recall	F1	AUC
K-nearest neighbor	0.214	0.691	0.737	0.486	0.586	0.776
Linear regression	0.240	0.707	0.685	0.642	0.663	0.777
Random forest	0.370	0.760	0.750	0.690	0.729	0.825
Protein- Sol	0.376	0.714	0.689	0.688	0.693	0.808
XGboost	0.385	0.756	0.748	0.690	0.718	0.829
Support vector machine	0.411	0.761	0.763	0.684	0.721	0.842
DeepSol	0.434	0.763	0.771	0.738	0.695	0.845
ProGAN	0.442	0.763	0.770	0.676	0.720	0.853
SeqVec	0.458	0.767	0.754	0.715	0.734	0.858
TAPE	0.461	0.764	0.774	0.710	0.730	0.856
LSTM	0.458	0.765	0.748	0.677	0.730	0.855
GraphSol	0.483	0.779	0.775	0.693	0.732	0.866
GraphSol (Ensemble)	0.501	0.782	**0.790**	0.702	0.743	0.873
**GATSol**	**0.517**	**0.791**	0.781	**0.745**	**0.763**	**0.882**

†Performance values for most methods are adopted from the original GraphSol paper, with the bolded values highlighting the best performance for a given situation

**Fig. 5:**
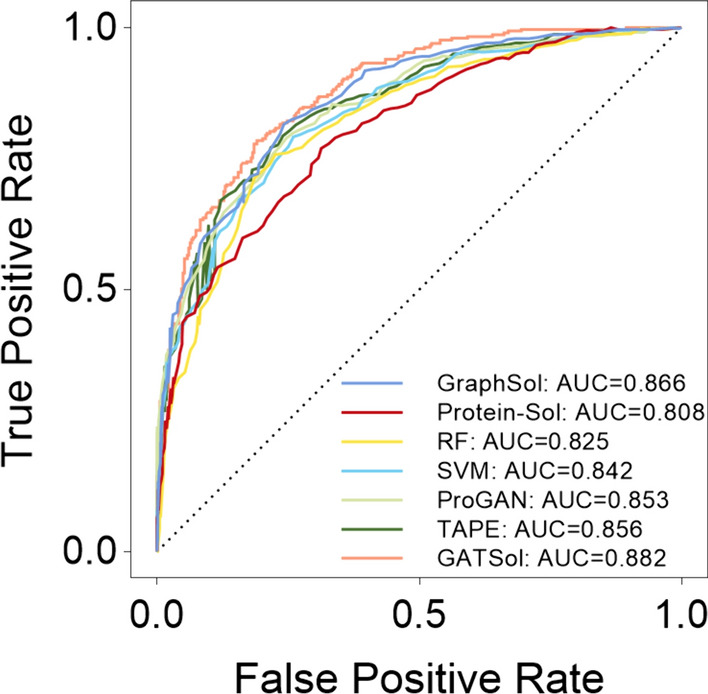
Performance comparison of GATSol with existing predictors of ROC and AUC on independent eSOL test dataset

Moreover, the GATSol model streamlines the proteolysis prediction process compared to GraphSol, making it more practical for real-world applications. In Fig. 5, when observing the ROC curves of the seven methods, it becomes evident that most of our model's curves occupy the upper echelons. This observation is a further testament to our model's superior performance in proteolysis prediction.

To underscore the versatility of the GATSol model further, we conducted a rigorous evaluation on the same independent test set of *S. cerevisiae*, akin to the procedure employed by GraphSol. Initially, the original *S. cerevisiae* test set contained 447 solubility-accessible proteins. After eliminating internal redundancy and redundancy concerning the training set, following the criteria of 30% global identity and an E-value of ≤ 1e−6, we were left with 368 proteins, denoted as *S. cerevisiae*_368. We also used the same independent test set featuring 108 proteins as GraphSol.

GATSol model's performance was rigorously assessed on the independent test set *S. cerevisiae*_368, yielding a commendable R^2^ value of 0.361. Furthermore, to facilitate a robust comparison with other models, a parallel evaluation was conducted on *S. cerevisiae*_108. The results, as detailed in Table 2, unveil the GATSol model's outstanding prowess, reflected by its remarkable R^2^ value of 0.424., marking an impressive improvement of more than 18.4% in contrast to the previously top-performing GraphSol, and an impressive 14% improvement compared to GraphSol (ensemble). It's worth noting that all other methods returned lower R^2^ values on this dataset, potentially attributable to our model's prowess in discerning concealed relationships within a dataset characterized by low homology. This proficiency enhances the model's generalization capabilities, allowing it to excel in uncharted data territory.

**Table 2:** Performance comparison of GATSol with existing protein solubility predictors on the independent *S. cerevisiae*_108 test dataset†

Solubility predictors	R^2^
**GATSol**	**0.424**
GraphSol (ensemble)	0.372
GraphSol	0.358
ccSol	0.302
Protein-Sol	0.281
CamSol	0.160
DeepSol	0.090
ProGANb	0.084

†Performance values for most of the methods are adopted from the original GraphSol paper, with the bolded values indicating the best performance for a given situation

## Conclusion

This study introduces an innovative method, the GATSol model, for protein solubility prediction through the synergy of protein large language modeling and amino acid graph attention neural network modeling. GATSol outperforms most recently introduced models by about 0.517 and 0.424 of R^2^ when tests on the independent eSOL and the *S. cerevisiae* test datasets. One of the key elements of GATSol, compared to other models, is the use of protein graph based on ESM-1b model and the 3D protein structures predicted by Alphafold. It plays an important role in harnessing the power of graph neural networks to reveal three-dimensional features from in one-dimensional sequences. GATSol simplifies the entire prediction process by requiring only the input of protein sequences and the structures predicted by Alphafold, thus greatly simplifying the prediction process, making it easier to use and more efficient. We can expect GATSol to help prioritize highly soluble proteins, which ultimately reduces the cost and effort of the practical experimental work.

## Supplementary Information

Supplementary file 1: Figure S1. The distribution of sequence lengths and solubilities of the proteins used for training and testing. Table S1. 5-fold cross-validation results on the training set.

## Data Availability

All data and code are provided at https://github.com/binbinbinv/GATSol.
