# Supplementary material for: GATSol, an enhanced predictor of protein solubility through the synergy of 3D structure graph and large language modeling
Source: BMC Bioinformatics. 2024 Jun 1;25:204. doi: 10.1186/s12859-024-05820-8 (PMC11549816; doi:10.1186/s12859-024-05820-8)
Supplement: Supplementary file 1 — Figure S1. The distribution of sequence lengths and solubilities of the proteins used for training and testing. Table S1. 5-fold cross-validation results on the training set. [file 12859_2024_5820_MOESM1_ESM.pdf]

Supporting information

Figure S1. The distribution of sequence lengths and solubilities of the proteins used for training and testing. A: Distribution of solubility for each sample in the dataset.B: Distribution of eSol data used for training across different sequence length intervels.C: Distribution of eSol data used for testing across different sequence length intervels.D: Distribution of S.cerevisiae data used for testing across different sequence length intervels.

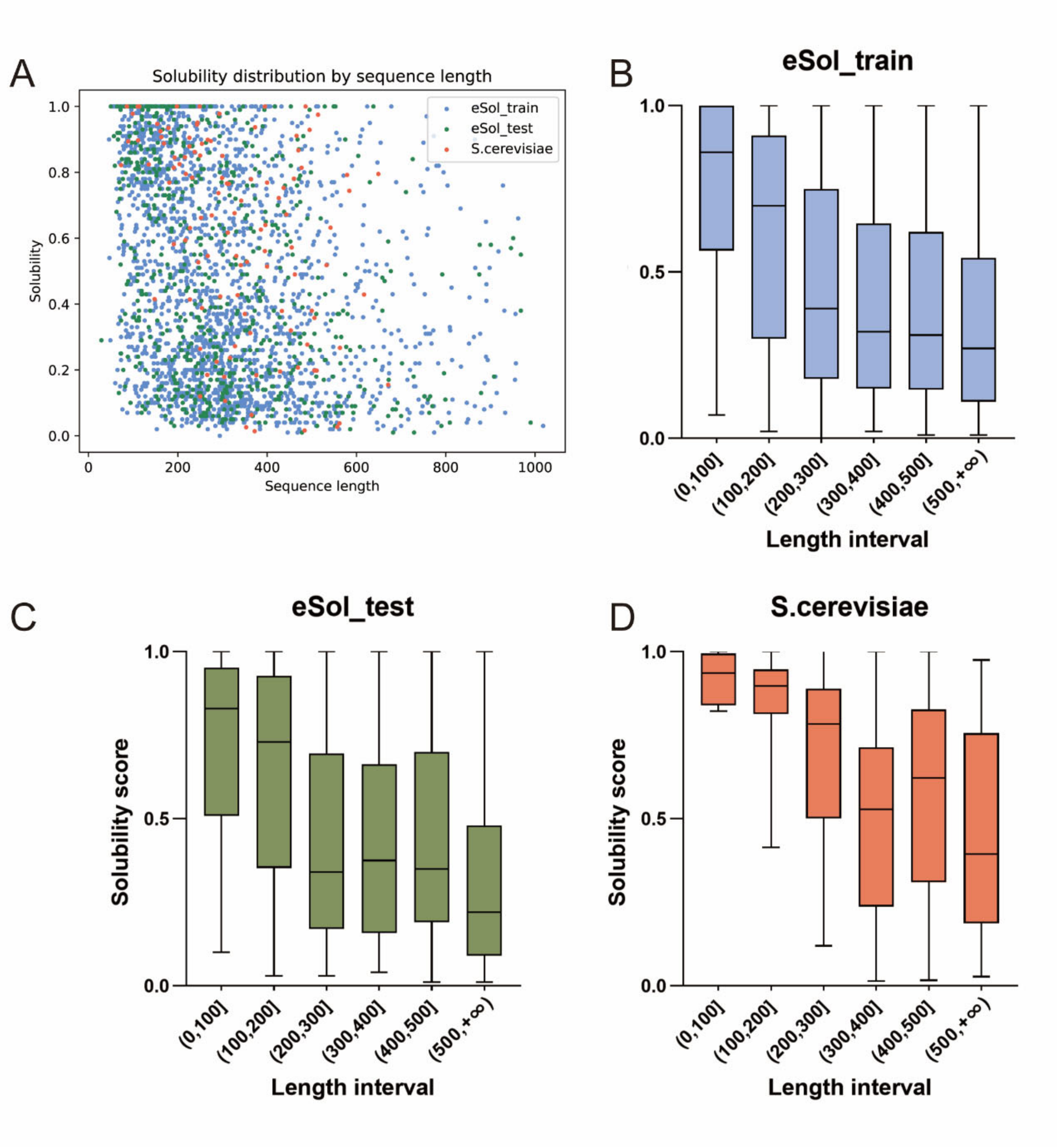

Table S1. 5-fold cross-validation results on the training set

①Distance threshhold of the pair of amino acid

Distance threshhold of the pair of amino acid（Å）	R^2
6	0.415±0.0155
7	0.411±0.0155
8	0.417±0.0280
9	0.412±0.0220
10	0.420±0.0290
11	0.407±0.0170
12	0.402±0.0085
13	0.407±0.0125
14	0.409±0.0160
15	0.402±0.0175

②Learning rate

Learning rate	R^2
0.002	0.403±0.043
0.0002	0.402±0.059
0.00002	0.404±0.056
0.000002	0.399±0.039
0.0000002	0.272±0.030
0.00000002	0.023±0.027

③Number of batch size

Number of batch size	R^2
1	0.415±0.042
2	0.414±0.048
4	0.416±0.054
8	0.415±0.046
16	0.414±0.042
32	0.416±0.045
64	0.415±0.046
128	0.410±0.044
256	0.414±0.040
512	0.412±0.045

④Number of hidden channels

Number of hidden channels	R^2
32	0.273±0.023
64	0.321±0.012
128	0.377±0.061
256	0.400±0.042
512	0.414±0.047
1024	0.420±0.055

⑤Number of attention heads

**Number of attention heads**	**R^2**
1	0.305±0.026
2	0.350±0.048
3	0.376±0.028
4	0.382±0.029
5	0.397±0.017
6	0.402±0.039
7	0.40±0.026
8	0.406±0.025
9	0.405±0.035
10	0.413±0.035
11	0.407±0.045
12	0.408±0.030
13	0.410±0.025
14	0.408±0.038
15	0.411±0.031
16	0.416±0.028
17	0.416±0.033
18	0.416±0.029
19	0.412±0.018
20	0.411±0.025
